# Triphenylphosphonium-Conjugated Palmitic Acid for Mitochondrial Targeting of Pancreatic Cancer Cells: Proteomic and Molecular Evidence

**DOI:** 10.3390/ijms25126790

**Published:** 2024-06-20

**Authors:** Giuliana Siragusa, Jessica Brandi, Tristan Rawling, Michael Murray, Daniela Cecconi

**Affiliations:** 1 Department of Biotechnology, University of Verona, Strada le Grazie 15, 37134 Verona, Italy; giuliana.siragusa@univr.it (G.S.); jessica.brandi@univr.it (J.B.); 2 School of Mathematical and Physical Sciences, Faculty of Science, University of Technology Sydney, Sydney, NSW 2007, Australia; tristan.rawling@uts.edu.au; 3 Molecular Drug Development Group, Sydney Pharmacy School, Faculty of Medicine and Health, The University of Sydney, Sydney, NSW 2006, Australia; michael.murray@sydney.edu.au

**Keywords:** mitochondria, reactive oxygen species, palmitic acid, triphenylphosphonium, pancreatic cancer

## Abstract

Pancreatic ductal adenocarcinoma (PDAC)’s resistance to therapies is mainly attributed to pancreatic cancer stem cells (PCSCs). Mitochondria-impairing agents can be used to hamper PCSC propagation and reduce PDAC progression. Therefore, to develop an efficient vector for delivering drugs to the mitochondria, we synthesized tris(3,5-dimethylphenyl)phosphonium-conjugated palmitic acid. Triphenylphosphonium (TPP) is a lipophilic cationic moiety that promotes the accumulation of conjugated agents in the mitochondrion. Palmitic acid (PA), the most common saturated fatty acid, has pro-apoptotic activity in different types of cancer cells. TPP-PA was prepared by the reaction of 16-bromopalmitic acid with TPP, and its structure was characterized by ^1^H and ^13^C NMR and HRMS. We compared the proteomes of TPP-PA-treated and untreated PDAC cells and PCSCs, identifying dysregulated proteins and pathways. Furthermore, assessments of mitochondrial membrane potential, intracellular ROS, cardiolipin content and lipid peroxidation, ER stress, and autophagy markers provided information on the mechanism of action of TPP-PA. The findings showed that TPP-PA reduces PDAC cell proliferation through mitochondrial disruption that leads to increased ROS, activation of ER stress, and autophagy. Hence, TPP-PA might offer a new approach for eliminating both the primary population of cancer cells and PCSCs, which highlights the promise of TPP-derived compounds as anticancer agents for PDAC.

## 1. Introduction

Pancreatic ductal adenocarcinoma (PDAC) is the most prevalent neoplastic disease of the pancreas, representing more than 90% of all pancreatic malignancies. PDAC ranks as the seventh most common cause of cancer-related mortality, with projections indicating it will climb to second place by 2030 [1]. The dismal prognosis associated with PDAC stems from late diagnosis, early metastasis, resistance to standard therapies, and frequent recurrence of tumors. Emerging evidence points to pancreatic cancer stem cells (PCSCs) as key drivers of these challenges [2]. These cells, a small subset within tumors, exhibit traits such as self-renewal, anchorage-independent growth, prolonged proliferation, and resistance to radiation and chemotherapy. Current efforts in PDAC treatment development emphasize targeting PCSCs. Research in this area focuses on understanding the mechanisms that maintain PCSC stemness as well as their metabolic and epigenetic profiles [3]. In particular, energetic metabolism and mitochondrial function play fundamental roles in phenotype maintenance and the spreading of PCSCs. Therefore, mitochondria-targeting agents might be used to reduce PCSC viability and hamper their propagation.

Growing evidence suggests that various categories of naturally derived and synthetic lipids, including fatty acids (FAs) of varying chain length and unsaturation, possess the potential to be developed as anticancer agents, exerting their effects, at least partially, at the level of the mitochondrion [4]. For instance, palmitic acid (PA, C16:0), the most common saturated fatty acid in the human diet, has emerged as a promising anti-tumor agent, demonstrating efficacy against various malignancies [5]. PA induces cell apoptosis through the mitochondrial pathway, a process amplified by the increase in reactive oxygen species (ROS) within cells [6]. Additionally, PA induces programmed cell autophagic death and cell cycle arrest and also inhibits cell migration, invasion, and angiogenesis while simultaneously boosting the effectiveness of chemotherapy and diminishing side effects. It has also been reported that PA acts on critical pathways involved in cancer cell behavior, such as the phosphatidylinositol 3-kinase (PI3K)/protein kinase B (Akt), the endoplasmic reticulum (ER), B Cell Lymphoma-2 (Bcl-2), and p53, among others [5].

Triphenylphosphonium (TPP)-based compounds are delocalized lipophilic cations that accumulate in mitochondria and act as potent inhibitors of mitochondrial function in cancer cells and cancer stem cells (CSCs) [7]. The TPP+ moiety has a significant effect on mitochondria, causing respiratory chain and Krebs cycle dysfunction [8]. Interestingly, compounds carrying a TPP+ moiety preferentially accumulate in the cancer cell mitochondrion to provide selective targeting of cancer cells and CSCs over normal cells. In addition, when TPP+ is conjugated with a bioactive compound, the cytotoxic effects of the latter may be either potentiated or modulated [9]. For this reason, TPP+ has been widely used in the design of mitochondria-targeted compounds for different applications (i.e., anticancer, antifungal, antioxidative, etc.) [10,11].

Therefore, considering the strategic importance of TPP-based compounds to target cancer cells and CSCs, in this study, we evaluated the anticancer effect of the novel TPP-conjugated PA (TPP-PA) on an in vitro model of PDAC cells. We used a label-free quantitative mass spectrometry (MS) approach to compare the proteomes of TPP-PA-treated and untreated parental PDAC cells and PCSCs, detecting dysregulated proteins and pathways. The mitochondrial membrane potential, cardiolipin content, total intracellular ROS, and lipid peroxides were also evaluated after TPP-PA treatment. Additionally, immunoblotting was performed to assess markers of ER stress and autophagy. The results obtained allow us to highlight the anti-proliferative effect on PDAC cells and clarify, from a molecular point of view, the mechanism of anti-tumor action of the novel agent TPP-PA.

## 2. Results and Discussion

### 2.1. Synthesis of TPP-Conjugated Palmitic Acid

**Figure 1:**
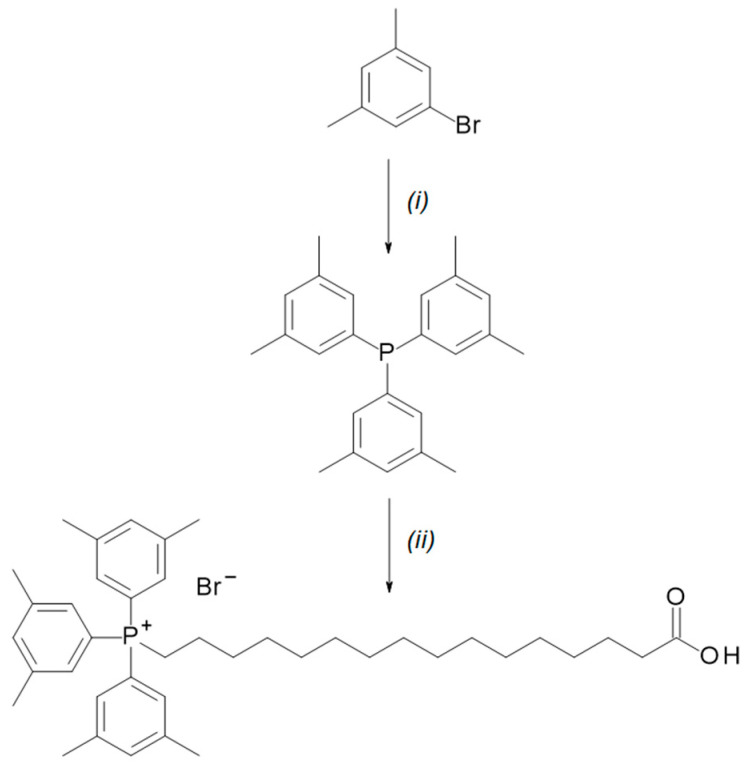
Synthesis of TPP-PA. Regents and conditions: (i) butyl lithium, tetrahydrofuran (THF), 0 °C, then PCl_3_. (ii) 16-bromopalmitic acid, 150 °C.

TPP-PA was synthesized in two steps (Figure 1). In the first step, 1-bromo-3,5-dimethylbenzene was reacted with butyl lithium, and phosphorous trichloride was added to the lithium salt. The displacement of the chloride groups provided tris(3,5-dimethylphenyl)phosphane, which was partially purified by recrystallization. In the second step, the crude phosphane was heated with 16-bromopalmitic acid in a solvent-free reaction, and the precipitation of the product provided TPP-PA as a solid.

### 2.2. TPP-PA Decreases the Viability of PCSCs and PDAC Cells

**Figure 2:**
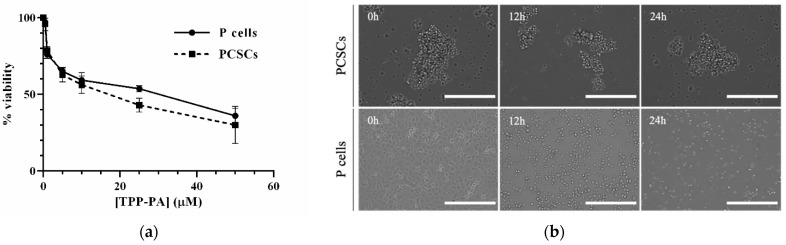
Cell viability and morphology of PANC-1 cells, including P and PCSCs, after TPP-PA treatment. (**a**) Cell viability after treatment with different concentrations of TPP-PA for 24 h. (**b**) Morphology of cells after treatment with 18 µM TPP-PA for 12 and 24-h. 10× magnification, scale bar = 400 μm.

To evaluate the effect of TPP-PA on the proliferation of PANC-1 parental (P) and PCSCs, the percentage of cell growth was measured after 24 h of treatment with increasing concentrations of TPP-PA ranging from 0 μM to 50 μM (Figure 2a). The cells treated with TPP-PA showed a dose-dependent inhibition of viability, with IC50s of 31 and 18 μM in P and PCSCs, respectively. Having assessed the IC50s, the TPP-PA concentration of 18 μM, corresponding to the IC50 in PCSCs, was selected for subsequent assays because PCSCs are important chemoresistant cells in PDAC. The effect of TPP-PA on the morphology of PANC-1 P and PCSCs was also evaluated using microscopy (Figure 2b). Treatment with 18 μM TPP-PA for 12 and 24 h did not influence the potential to generate tumorspheres. Conversely, P cells progressively acquired a more rounded shape and decreased cell-to-cell contact; these features are typical of cellular death.

### 2.3. Proteomics Unveils Changes in Mitochondrial and Stress-Related Proteins

**Figure 3:**
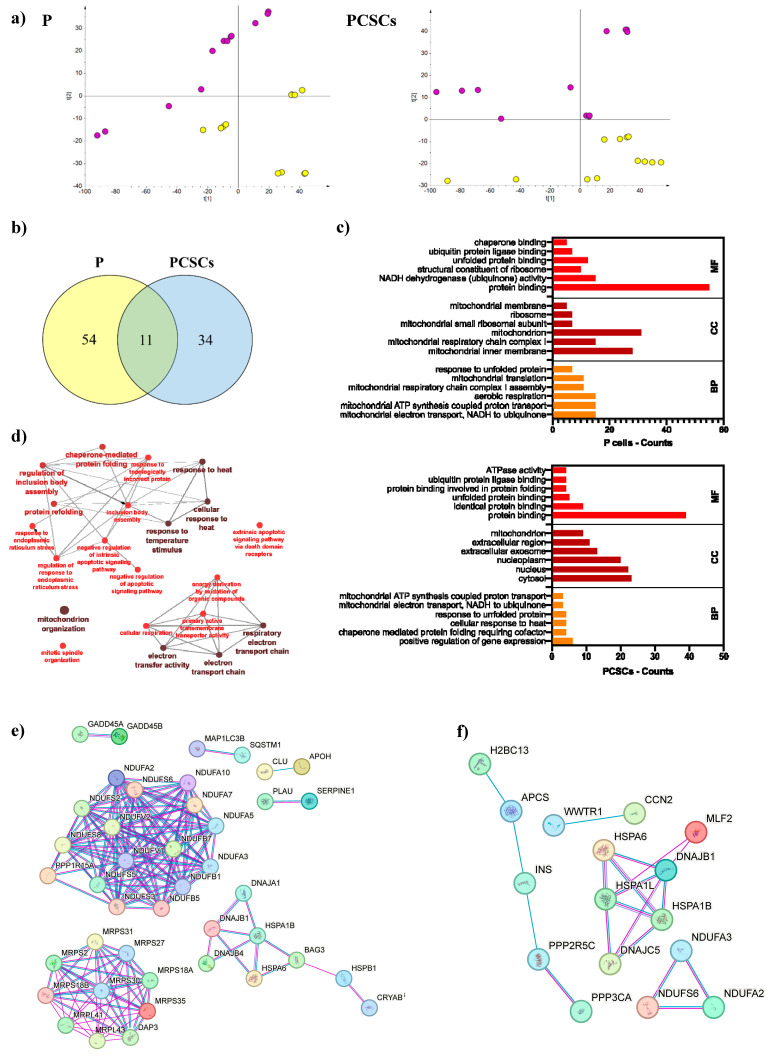
Proteomics results of PANC-1 cells, including P and PCSCs, after 18 µM TPP-PA treatment for 24 h. (**a**) Score plot of PCA analysis to overview classification trend of proteome profiles of treated (yellow circles) and untreated (purple circles) cells. (**b**) Venn diagram representing the overlap among DEPs identified by LC-MS/MS analysis. (**c**) GO enrichment analysis of DEPs retrieved using DAVID. The six most significantly (*p* < 0.05) enriched GO terms in molecular function (MF), cellular component (CC), and biological process (BP) branches are presented. (**d**) Visualization of biological processes characterizing the DEPs of treated cells obtained by Cytoscape. The node size is proportional to the number of proteins, and the node color depicts the enrichment significance (ranging from red = *p*-value < 0.05, to dark red = *p*-value < 0.005, and dark brown = *p*-value < 0.0005). (**e**,**f**) STRING-based interaction analysis of DEPs in treated P cells (**e**) and treated PCSCs (**f**). The circles represent the identified proteins, and the edges represent protein–protein interactions. Blue lines represent known interactions from curated databases, pink lines represent experimentally determined interactions, purple lines indicate that protein homologs are found interacting in other organisms.

To obtain information about the modulation induced by TPP-PA at the protein level, three biological replicates of P and PCSCs were treated for 24 h with either 18 μM TPP-PA or 0.1% DMSO and subjected to proteomic analysis. By high-resolution accurate-mass Orbitrap MS, we identified a total of 3218 and 3195 proteins in P and PCSCs, respectively (Table S1). The principal component analysis (PCA) indicated that samples from different groups were well separated (Figure 3a). Regarding the protein dataset in the P cells, the first and second components accounted for 43.8% and 17.7% of the overall variance, respectively, while in the protein dataset from the PCSCs, the first and second components accounted for 67% and 16.6% of the overall variance, respectively. This suggests that there is sufficient variation between the treated and untreated cells, increasing the confidence that interesting protein modulations can be found in these datasets. Based on the proteomic analysis, differentially expressed proteins (DEPs) were detected between the untreated and treated cells of two phenotypes, P and PCSCs, respectively (Table S1). Among these DEPs, 28 were significantly up-regulated and 37 were significantly down-regulated in the TPP-PA-treated P cells. In contrast, 24 DEPs were significantly up-regulated and 21 DEPs were significantly down-regulated in the TPP-PA-treated PCSCs. In follow-up studies, Venn diagram analysis showed that 54 and 34 DEPs were selectively expressed in P and PCSCs, respectively, while 11 were common to both cell types (Figure 3b).

GO enrichment analysis illustrated that the DEPs of P cells (Figure 3c) are primarily mitochondrial, belonging to the inner membrane, respiratory chain, and mitochondrial ribosomes. Mitochondrial ribosomal proteins (MRPs) facilitate the translation of several proteins of the OXPHOS complex, and preclinical studies have indicated that their inhibition is associated with anticancer activity [12]. In the present study, 12 MRP subunits were down-regulated in the TPP-PA-treated P cells (Table S1). The GO annotation also revealed that most of the DEPs of the P cells were involved in mitochondrial electron transport and mitochondrial ATP synthesis processes and largely played protein-binding and NADH dehydrogenase activities. On the other hand, in the TPP-PA-treated PCSCs (Figure 3c), DEPs were mostly cytosolic or nuclear, being implicated in the positive regulation of gene expression, responses to heat, and unfolded proteins, with molecular functions that mainly concern the binding of proteins, including unfolded ones. Together, these data suggest that treatment with 18 μM TPP-PA for 24 h produces distinct effects in the different cell types, including mitochondrial targeting in P cells and dysregulated responses to cellular stress and protein unfolding in PCSCs.

To further investigate the main biological processes related to TPP-PA treatment, a Cytoscape analysis was performed (Figure 3d). Overall, the DEPs of P and PCSCs showed a significant link with different biological processes, including, among the most statistically significant, “mitochondrion organization”, “respiratory electron transport chain”, and “cellular response to heat”, which shared DEPs with “protein folding” and “response to endoplasmic reticulum stress”.

Moreover, the network interaction between DEPs was analyzed using the STRING database. As shown in Figure 3e, the PPI network derived from DEPs in the TPP-PA-treated P cells exhibited a high average node degree, as indicated by two large groups of interacting proteins: one consisting of MRPs and the other of NADH:ubiquinone oxidoreductase subunits (NDUFs). In addition, a smaller subnetwork of cytoplasmic chaperones that are implicated in the response to cellular stresses, such as heat shock proteins (HSPs) and DNAJ proteins (DNAJs), was identified. In contrast, the PPI network generated for the DEPs in the treated PCSCs (Figure 3f) displayed a low average node degree, consistent with limited interconnectivity among proteins. Accordingly, only two small subnetworks were identified, one involving NDUFs and the other with six chaperones (HSPs and DNAJs). These findings further revealed that modulated proteins in the P cells that were treated with 18 μM TPP-PA primarily participated in mitochondrial processes. In contrast, the proteins that were modulated in the treated PCSCs, while involving NDUFs, seem to be predominantly linked to cellular stress.

In this regard, the proteomics data (Table S1) demonstrated that three subunits (i.e., NDUFA2, NDUFA3, and NDUFS6), which are NDUFs that are associated with complex I of the electron transport chain (ETC), were down-regulated in both P and PCSCs after TPP-PA treatment. Moreover, 12 additional NUDFs were selectively down-regulated in the treated P cells. It is noteworthy that NDUFs play pivotal roles in proliferation, resistance to cell death, and metastatic activity of cancer cells, and that their targeting has been shown to overcome chemoresistance in PDAC tumors [13]. The loss of NDUFs in tumor cells is implicated in cytotoxicity mediated by oxidative stress and occurs in concomitance with the induction of ROS and lipid peroxidation [14]. Therefore, the observed down-regulation of NUDFs is in line with mitochondrial targeting by TPP-PA [15].

Several DEPs that regulate cellular stress responses, including members of the Hsp-70 and DNAJ families of proteins (also termed Hsp-40), were up-regulated in both P and PCSCs (Table S1). Notably, it has been shown that mitochondrial ETC dysfunction activates the response to heat shock, which is mediated by oxidative stress [16]. Among the detected DEPs, the heat shock 70 kDa protein 1A (HSPA1A) is known to interact with mitochondrial cardiolipin, which may facilitate its translocation to sub-cellular compartments (such as mitochondria or endosomes), where it regulates membrane stabilization, autophagy, and apoptosis [17]. Other DEPs induced by TPP-PA in P and PCSCs included DNAJB1 and HSPA6, which are chaperones implicated in ER stress and the unfolded protein response (UPR) [18,19], as well as in autophagy [20]. DNAJB1 and HSPA6 are two of the most significant genes that are dependent on ATF3, a transcription factor induced by TPP-PA in PCSCs (Table S1), which belongs to the pro-apoptotic ATF4/CHOP/ATF3 arm of the UPR [21].

In accordance with this finding, the anterior gradient protein 2 homolog (AGR2), which is a member of the disulfide isomerase family of ER proteins that regulate protein folding, was down-regulated by TPP-PA in both P and PCSCs (Table S1). Agr2 is a proto-oncogene predominantly localized in the ER but is also secreted and implicated in the initiation of pancreatic cancer [22]. Indeed, Agr2 is increased in the plasma of pancreatic cancer patients, and secreted Agr2 plays a role in cell stemness, migration, and metastasis [23,24]. Notably, it has been demonstrated that intracellular and extracellular Agr2 protect PANC-1 cells from ER stress-induced apoptosis and that increased Agr2 promotes PANC-1 cell survival after gemcitabine treatment [25].

### 2.4. TPP-PA Targets the Mitochondrion and Induces Total ROS and Lipid Peroxidation

**Figure 4:**
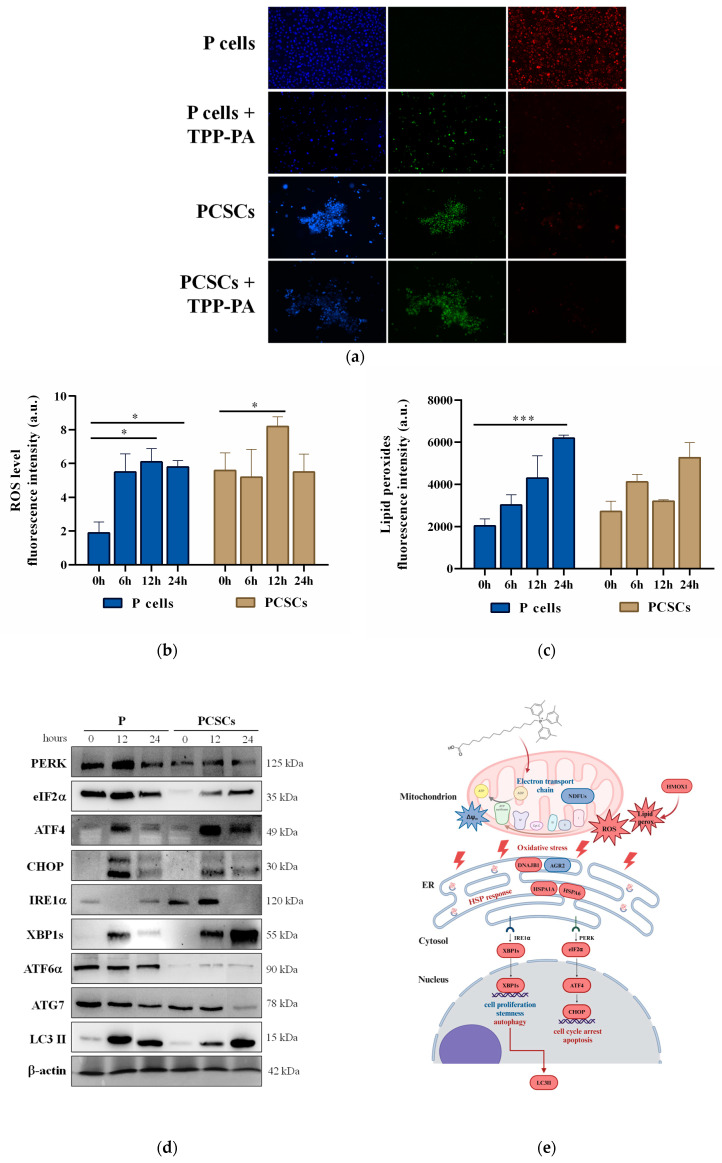
Assessment of mitochondrial membrane potential, ROS production, ER stress, and autophagy in PANC-1 cells, including P and PCSCs, after 18 µM TPP-PA treatment. (**a**) JC-1 staining image after 24 h of treatment with TPP-PA. Red fluorescence indicates preserved ΔΨm, while green fluorescence indicates membrane depolarization. (**b**) ROS production assessed by detection of DCF fluorescence (arbitrary units) in microplate assays after 0, 6, 12, and 24 h of TPP-PA treatment. Data are presented as mean ± standard deviation (n = 3). One asterisk (*) indicates *p* < 0.05. (**c**) Lipid peroxidation assessed by FACS analysis of C11-BODIPY fluorescence after 0, 6, 12, and 24 h of TPP-PA treatment. Data are presented as mean ± standard deviation (n = 3). Three asterisks (***) indicate *p* < 0.001. (**d**) Immunoblot analysis of ER stress- and autophagy-related factors after TPP-PA treatment for 0, 12, and 24 h. Beta-actin served as a loading control. The blots were cropped to focus on the specific proteins indicated. (**e**) Mechanisms involved in in vitro anticancer activity of TPP-PA against pancreatic cancer cells. In red or blue (font and shape color) are the proteins and processes that are induced or repressed, respectively. Created with BioRender.com.

To further elucidate the molecular mechanism of the anticancer action of TPP-PA, we tested the hypothesis that oxidative stress may be induced. Numerous studies have shown a positive relationship between the mitochondrial membrane potential (ΔΨ) and the production of ROS [26]. In the present study, JC-1 dye was employed to evaluate the ΔΨ in P and PCSCs after treatment with 18 μM TPP-PA for 24 h. As shown in Figure 4a, compared with the control, the ΔΨ was decreased in the P cells treated with TPP-PA, as reflected by an increase in JC-1 monomer (which appeared as green fluorescence, indicating mitochondrial depolarization) and a decrease in JC-1 polymer (which appeared as red fluorescence, indicating mitochondrial hyperpolarization). In contrast, PCSCs showed low ΔΨ both before and after TPP-PA treatment, as indicated by similar green fluorescence. These findings indicate that the ΔΨ is altered in P cells but not in PCSCs by TPP-PA. In addition, the observation that the ΔΨ was low in the untreated PCSCs suggests that depolarization could play a key role in the maintenance of cancer stem cells in an undifferentiated state [27]. Next, because the loss of cardiolipin has been linked to the mechanism of action of other mitochondrion-targeted fatty acids [28], as well as to mitochondrial depolarization, we measured cardiolipin levels in the TPP-PA-treated cells. The content of cardiolipin detected by a fluorescent probe was unchanged in both P and PCSCs following TPP-PA treatment (Table 1), suggesting that the alterations in the ΔΨ are not linked to the loss of cardiolipin. However, changes in the composition or oxidation of individual cardiolipin sub-species cannot be excluded.

Given that TPP-PA causes a depolarization of the ΔΨ in P cells and dysregulates the electron transport proteins in both P and PCSCs (as indicated by the proteomics results, Table S1), we tested whether increased ROS production could mediate the loss of viability in TPP-PA-treated P cells and PCSCs (Figure 4b). Using the redox-responsive H2DCFDA probe, ROS formation was increased to about three-fold that of the control in the treated P cells at 6 h and was maintained after 12 and 24 h of treatment. On the other hand, in the treated PCSCs, the increase in ROS production was lower, i.e., about 1.5-fold that of the control, which occurred only after 12 h of treatment and was followed by a return to baseline levels after 24 h of treatment. These results are in line with the modulation of the ΔΨ, which was highlighted at 24 h for P cells but not for PCSCs. Furthermore, the higher basal ROS levels in PCSCs compared to P cells are consistent with the role of ROS as a second messenger that regulates proliferation and self-renewal in cancer stem cells (CSCs) [29]. Moreover, these findings further indicate the distinct effects of 18 μM TPP-PA on the two cell phenotypes, which could be related to differences in metabolic characteristics and antioxidant defense mechanisms. Indeed, it is increasingly evident that the anti-tumorigenic versus pro-tumorigenic effects of ROS have threshold levels and are cell-specific [30].

The findings from the proteomics analysis (Table S1) indicated that the DEPs that play a role in ferroptosis, such as heme oxygenase 1 (HMOX1) [31], were up-regulated in both the P cells and PCSCs after TPP-PA treatment. Several other DEPs that are associated with ferroptosis, including ATP synthase F(0) complex subunit C3 (ATP5MC3) [32], sequestosome-1 (SQSTM1) [33], and protein C-ets-2 (ETS2), which is a potent transactivator of HMOX1 [34], were also increased in the treated P cells, while the WW domain-containing transcription regulator protein 1 (WWTR1) [35] and the already mentioned ATF3 [36] were up-regulated in the treated PCSCs. It is noteworthy that PA induces ferroptosis via ER stress in colon cancer cells [37].

Therefore, taking into consideration that the DEPs and the elevated levels of intracellular ROS may increase lipid peroxidation and promote ferroptosis, we examined lipid peroxidation using C-11 BODIPY staining (Figure 4c). Intriguingly, after 6 h of TPP-PA treatment, lipid peroxidation was increased to approximately 1.5-fold that of the control. However, while peroxidation was further increased in the P cells by 12 and 24 h of TPP-PA treatment, lipid peroxidation was more variable in the treated PCSCs. These findings reveal distinct modulation of lipid peroxidation in the two subsets of treated PANC-1 cells (i.e., P and PCSCs) and further underscore the differences and heterogeneity in the activation of metabolic pathways and the response to external stress or stimuli. Despite these differences, the overall increase in total ROS and lipid peroxidation produced by TPP-PA treatment was evident.

### 2.5. TPP-PA Activates the CHOP and XBP1s Branches of ER Stress Pathway and Autophagy

The proteomics data (Table S1) showed that the expression of HSPA1A, DNAJB1, and HSPA6 was increased in both P and PCSCs by TPP-PA; as mentioned, these proteins are implicated in ER stress and the UPR. Consistent with this finding, the growth arrest and DNA damage-inducible proteins GADD45-alpha (GADD45A) and GADD45-beta (GADD45B), which drive cells toward UPR-induced apoptosis [38], were also up-regulated in the treated P cells. Further, hornerin (HRNR), which activates ER stress mediated through the IRE1α/XBP1 pathway [39], was also up-regulated in the TPP-PA-treated PCSCs. Therefore, in view of reported relationships between ROS, mitochondria, ER stress, UPR, and the induction of autophagy [40], we explored the effect of TPP-PA on important regulators of ER stress and autophagy.

The immunoblotting data indicated (Figure 4d) that the treatment of P cells and PCSCs with TPP-PA modulates UPR pathways mediated by the sensor proteins PKR-like ER signaling kinase (PERK) and inositol-requiring enzyme 1 (IRE1) but not activating transcription factor 6 (ATF6). Indeed, the expression of eukaryotic initiation factor-2α (eIF2α), activating transcription factor 4 (ATF4), and C/EBP homologous protein (CHOP), which are ER stress markers downstream from PERK, was increased in treated P and PCSCs. Furthermore, the lack of expression of IRE1α in the TPP-PA-treated P cells at 12 h and PCSCs at 24 h seemed to be counterbalanced by overexpression of the downstream spliced form of X-box binding protein-1 (XBP1s). Increased expression was evident in both cell types at 12 h and appeared optimal in PCSCs at 24 h. Interestingly, the PERK and IRE1 branches of the UPR have been shown to regulate CSCs and tumor development, and it has been reported that enhanced expression of XBP1s reduces proliferation and stemness in cancer cells by cross-activating PERK-eIF2α signaling [41]. Moreover, elevated expression of CHOP can induce cell cycle arrest and promote apoptosis [42], and XBP1s can activate autophagy by promoting the conversion of LC3 I to LC3 II [43].

In accordance with these observations, the proteomics results showed that the microtubule-associated protein 1A/1B light chain 3B (MAP1LC3B) was up-regulated in P and PCSCs after TPP-PA treatment (Table S1). MAP1LC3B encodes for the light chain 3B (LC3B), which is an established marker of autophagy that is involved in autophagosome biogenesis. Recently, LC3B has also been linked to cardiolipin-mediated degradation of damaged mitochondria by autophagy (mitophagy) in a process where cardiolipin acts as a specialized receptor for LC3 proteins [44]. Therefore, in order to gain a deeper insight into the potential impact of the discussed ER stress modulations on the autophagic pathway, the expression of autophagy-related (ATG) proteins, specifically ATG7 and light chain 3B (LC3B), was further examined (Figure 4d). The immunoblotting data indicated that TPP-PA treatment of P cells and PCSCs leads to down-regulation of ATG7 and up-regulation of LC3B type II. Atg7 is required for the formation and expansion of autophagosomes, which it triggers by initiating the conjugation of Atg12 to Atg5 and of LC3 type I to phosphatidylethanolamine, generating LC3 type II. However, it has also been reported that reduced degradation of Atg7 is implicated in the resistance of PDAC cells to gemcitabine [45] and that knockdown of ATG7 suppresses the proliferation and metastasis of PDAC cells [46]. On the other hand, PA strongly enhanced the expression of the LC3 type II protein [47] and induced autophagy through the ER stress-dependent pathway [48]. Overall, these results corroborate the induction of LC3B, as indicated by the proteomic analysis, and implicate autophagy in the mechanism of action of TPP-PA. The results also showed the induction of the type II form of LC3B and indicated that its formation does not imply the activation of the Atg7 pathway [49]. Together, the present findings suggest that the mechanisms by which TPP-PA inhibits the growth of PDAC cells (Figure 4e) involve mitochondrial targeting and modulation of the function of the endoplasmic reticulum and nucleus and implicate oxidative stress, activation of the UPR response, and the induction of autophagy.

## 3. Materials and Methods

### 3.1. Materials

Unless otherwise stated, reagents and anhydrous solvents were obtained from Sigma Aldrich (St. Louis, MO, USA). The PANC-1 cell line was provided by Professor Ilaria Dando, Biological Chemistry Section, Department of Neuroscience, Biomedicine, and Movement Sciences, University of Verona (Italy). The primary antibodies anti-PERK (sc-377400), anti-eIF2α (sc-133132), anti-CHOP (sc-7351), anti-ATF6α (sc-166659), and anti-ATG7 (sc-376212), as well as the secondary antibodies anti-mouse IgG-HRP (sc-516102) and anti-rabbit IgG-HRP (sc-2357), were purchased from Santa Cruz Biotechnology (Dallas, TX, USA). Anti-ATF4 (11815), anti-IREα (3294), and anti-LC3B (3868) were from Cell Signalling Technology (Arundel, QLD, Australia). Anti-XBP1 (GTX102229) was from Genetex (Irvine, CA, USA). C11-BODIPY (581/591) dye was from Thermo Fisher Scientific (Waltham, MA, USA; catalog number D3861), while the Cardiolipin Assay Kit was purchased from Sigma-Aldrich (catalog number MAK362).

### 3.2. Synthesis and Characterization of 15-Carboxypentadecyl-tris(3,5-dimethylphenyl) Phosphonium Bromide (TPP-PA)

1-bromo-3,5-dimethyl-benzene (4.00 g, 21.6 mmol) was dissolved in anhydrous THF (20 mL) at −78 °C, and n-butyllithium (2 M in cyclohexane, 25.9 mmol) and PCl3 (7.1 mmol) were added to the solution. The solution was stirred at 0 °C for 6 h, and then the reaction was quenched with brine (30 mL). The product was extracted with diethyl ether (2 × 30 mL), and the organic layer was dried over MgSO_4_ and filtered. The solvent was removed in vacuo, and the product was recrystallized from hot ethanol (20 mL). The crude tris(3,5-dimethylphenyl)phosphane and 16-bromopalmitic acid (2.39 g, 7.1 mmol) were stirred at 150 °C for 18 h. The mixture was cooled to room temperature, and the resulting solid was dissolved in a minimal volume of dichloromethane. Diethyl ether was added until precipitate formed, and the solid was collected by filtration and washed with diethyl ether to yield 2.08 g (43%) of TPP-PA.

^1^H NMR (500 MHz, CDCl_3_): δ 7.53 (s, 3H), 7.31 (s, 3H), 7.28 (s, 3H), 3.50–3.40 (m, 2H), 2.39 (s, 18H), 2.36 (t, J = 7.5 Hz, 2H), 1.62–1.52 (m, 6H), 1.32–1.14 (m, 20H). ^13^C NMR (125 MHz, CDCl_3_): δ 177.8, 146.4 (J = 3 Hz), 142.9 (J = 9 Hz), 134.6 (J = 11 Hz), 134.4 (J = 11 Hz), 128.5 (J = 13 Hz), 112.9 (J = 84 Hz), 34.5, 30.6 (J = 16 Hz), 29.4, 29.3, 29.2, 29.1, 29.1, 28.9, 24.9 (J = 5 Hz), 24.8, 24.7, 24.4, 22.6 (J = 4 Hz), 21.5. HRMS (ESI) *m*/*z* [M]+ calcd for C_40_H_58_PO_2_ 601.4163; found 601.4159.

### 3.3. Cell Culture

The human PDAC cell line PANC-1, referred to as parental (P) cells, was cultured in RPMI 1640 medium supplemented with 10% fetal bovine serum, 2 mM glutamine, and 50 μg/mL gentamicin sulfate (Gibco, Thermo Fisher Scientific) at 37 °C in a humidified 5% CO_2_ incubator. PCSCs were obtained as previously described [50] using the “CSC medium” (i.e., DMEM/F-12, B27, 1 g/L glucose, penicillin/streptomycin, heparin, epidermal growth factor, and fibroblast growth factor) for 2 weeks. PCSCs were then passed through a cell strainer (>40 μm) to collect tumorspheres.

### 3.4. Cell Treatment and Viability Assay

The ability of TPP-PA to decrease the viability of PANC-1 P cells and derived PCSCs was assessed in three independent experiments using the Cell Titer Fluor^TM^ Cell Viability Assay (Promega; Madison, WI, USA) according to the manufacturer’s instructions. Cells were seeded in 96-well plates (1 × 10^4^ cells per well) and allowed to adhere (in the case of P cells) or to re-acquire the tumorsphere phenotype (in the case of PCSCs) overnight. The cells were then treated at various concentrations of TPP-PA, ranging from 0 to 50 µM, for 24 h. Control cells were treated with 0.1% DMSO. The fluorescence was measured at excitation/emission wavelengths of 380/505 nm using a microplate reader (Tecan Infinite PRO 200). The IC50 dose–response curves for both P cells and PCSCs were defined as the minimum compound concentration required for 50% cell death (relative to vehicle control).

### 3.5. Morphological Analysis

PANC-1 P cells and PCSCs were seeded into 6-well plates and treated the following day with 18 μM TPP-PA for 12 and 24 h. Control cells were treated with 0.1% DMSO. TPP-PA-treated and control cells were imaged at times 0, 12, and 24 h after the treatment using the EVOS FL Imaging System (Thermo Fisher Scientific). Only cell clusters >40 μm were considered tumorspheres. Images were acquired using a 10× objective lens.

### 3.6. Sample Preparation and LC-MS/MS-Based Proteomic Analysis

Three biological replicates of P and PCSCs were treated for 24 h with either 18 μM TPP-PA or 0.1% DMSO as a control. After 24 h, cells were pelleted, washed twice with 1× cold PBS, and then lysed with RIPA buffer (150 mM NaCl, 1.0% IGEPAL^®^ CA-630, 0.5% sodium deoxycholate, 0.1% SDS, 50 mM Tris, pH 8.0) supplemented with 1× protease phosphatase inhibitor cocktail.

Protein extraction, quantification, and digestion were performed as already reported [51].

Tryptic peptide samples (1 μg each) underwent label-free LC-MS/MS analysis using an Orbitrap Fusion Lumos Tribrid MS (Thermo Fisher Scientific) combined online with an Ultimate 3000 nano-UHPLC equipped with an EASY-Spray PepMAP RSLC C18 column (Thermo Fisher Scientific). Peptide elution was achieved using a gradient from 4% to 50% acetonitrile over a 90 min period. In MS acquisition, the instrument was set over the *m*/*z* range 375–1500 Da, and the resolution was 120,000 (at 200 *m*/*z*). The MS/MS resolution was set to 50,000 (at 200 *m*/*z*), selecting for fragmentation the precursor ions with intensities exceeding 3.0exp4 and charges between +2 and +5, with a dynamic exclusion of 45 s. Blank samples (75% ACN in water) and a QC sample (HeLa lysate digest) were injected every four samples during acquisition.

The raw MS data were processed using Proteome Discoverer software (v2.5) with a mass tolerance of 10 ppm for MS1 and 0.02 Da for MS2. The generated peptide masses were searched against the UniProt protein sequence database using the following settings: trypsin digestion, maximum of two missed cleavages, cysteine carbamidomethylation as fixed modification, methionine oxidization, and protein N-terminal acetylation as variable modifications. Confidence in identification was assessed by the Percolator algorithm with an FDR of 0.01 for proteins and peptides. Label-free quantification was based on at least two unique peptides for protein abundance calculation, using a pairwise ratio-based approach to detect protein ratios and a t-test background-based approach to calculate *p*-values. Proteins exhibiting a *p*-value of less than 0.05 and a fold change (FC) of ±1.3 were detected as statistically significantly modulated.

### 3.7. Bioinformatic Analysis

Gene ontology (GO) annotation by DAVID (https://david.ncifcrf.gov/, accessed on 4 August 2023), functional enrichment analysis using ClueGO, a Cytoscape plug-in (http://www.ici.upmc.fr/cluego/, accessed on 4 August 2023), and protein–protein interaction network analysis using the STRING (http://string-db.org) platform were performed as previously described [52]. Briefly, annotation was performed using the official gene symbols as identifiers, the Homo sapiens background, the GOTERM_DIRECT annotation categories, and a *p*-value < 0.05 as the cut-off criterion. The functional enrichment analysis was conducted to highlight the significantly enriched GO biological processes based on a corrected *p*-value < 0.05. The GO term restrictions were a minimum of 3 genes and covered a minimum of 3% of the genes.

Finally, protein–protein interaction network analysis was carried out by setting a medium confidence level (score 0.4) and considering only known interactions experimentally determined and based on a curated database.

### 3.8. Western Blotting

Total cell lysates were prepared and used for Western blots as already reported [14], incubating the PVDF membranes with anti-PERK (dilution 1:200), anti-eIF2α (dilution 1:200), anti-CHOP (dilution 1:200), anti-IRE1α (dilution 1:1000), anti-XBP1 (dilution 1:500), anti-ATF6α (dilution 1:200), anti-ATG7 (dilution 1:200), or anti-LC3B (dilution 1:1000) for 3 h at room temperature. After incubation, membranes were washed and then incubated with anti-rabbit or anti-mouse IgG-HRP for 1 h at room temperature. The immunocomplexes were visualized by enhanced chemiluminescence using a ChemiDoc MP imaging system (Bio-Rad; Hercules, CA, USA).

### 3.9. Mitochondrial Membrane Potential Assay

The JC-1 dye (5,5,6,6-tetrachloro-1,1,3,3-tetraethylbenzimidazolycarbocyanine iodide, Cayman Chemical) was used to evaluate mitochondrial membrane stability. First, P cells and PCSCs were seeded in 6-well plates at 3.5 × 10^5^ cells/well and cultured at 37 °C for 24 h. The next day, the cells in each well were treated with 0.1% DMSO or 18 µM TPP-PA and incubated for 24 h. Then, the cells in each well were incubated with JC-1 (37 °C, 25 min) according to the manufacturer’s instructions (Cayman Chemical). For nuclear staining, cells were also incubated with Hoechst 33342 (Thermo Fisher Scientific) at 37 °C for 10 min and then imaged using a fluorescence microscope (EVOS FL Imaging System, Thermo Fisher Scientific). The fluorescence of dimeric and monomeric JC-1 was imaged using excitation/emission wavelengths of 535/595 nm and 485/535 nm, respectively. In addition, Hoechst 33342 fluorescence was detected using excitation/emission wavelengths of 361/497 nm. Images were acquired using a 10× objective lens.

### 3.10. Total ROS Assay

Total intracellular ROS were measured based on the detection of the fluorescent product DCF (2′,7′-dichlorofluorescein) produced by the oxidation of H2DCFDA. PANC-1 P and PCSCs were seeded in duplicate on 96-well plates and treated with TPP-PA for 6, 12, and 24 h before incubation with 10 μM of DCF at 37 °C for 15 min. After the incubation medium was removed, cells were washed, and stained cultures were analyzed for green fluorescence. Fluorescence was then measured at excitation = 485 nm and emission = 535 nm using a microplate reader (Tecan Infinite^®^PRO 200), and values were reported as fold induction.

### 3.11. Lipid Peroxidation Assay

Lipid peroxidation was measured by C-11 BODIPY using flow cytometry. PANC-1 P and PCSCs were seeded in duplicate on 6-well plates, treated with TPP-PA for 6, 12, and 24 h, and then incubated with 1 μM C11-BODIPY (581/591) dye for 30 min at 37 °C. Cells were collected, washed, and suspended in PBS supplemented with 2% FBS and 2 mM EDTA (500 μL) for cytofluorimetric analysis (LSRFortessa X-20, Becton Dickinson; Franklin Lakes, NJ, USA). The C11-BODIPY dye underwent peroxidation by cellular ROS, resulting in a fluorescent product detected at a lower wavelength (510 nm, green), while the non-peroxidized form was detected at a higher wavelength (590 nm, red). An increase in the green/red fluorescence intensity ratio indicates the formation of lipid peroxides.

### 3.12. Cardiolipin Assay

Mitochondrial cardiolipin was measured using the Cardiolipin Assay Kit, which makes use of a proprietary probe that fluoresces in association with cardiolipin but not with any other lipids. The assay was performed according to the manufacturer’s instructions. Briefly, cells were seeded at 3 × 10^5^ cells in microplates and allowed to attach or acquire a tumorsphere phenotype for 24 h. Then cells were treated with TPP-PA and lysed, and the protein concentration of the cell lysate was determined to adjust the volume of the sample and the cardiolipin assay buffer. A total of 50 µL of the reaction mix, containing the fluorescent probe plus the CL assay buffer, was added to each well of the 96-well plate containing the samples and standards. After an incubation at room temperature for 5 min, fluorescence was recorded at excitation = 340 nm and emission = 480 nm using a microplate reader (Tecan infinite PRO 200).

### 3.13. Statistical Analysis

The data are expressed as the mean ± standard deviation from at least three independent experiments. Statistical significance was determined using the unpaired Student’s *t*-test (GraphPad Prism 5). A *p*-value of less than 0.05 was considered significant.

## 4. Conclusions

In conclusion, a novel triphenylphosphonium-conjugated analogue of palmitic acid (TPP-PA) was synthesized and characterized. Its structure was confirmed by NMR and HRMS, and its anticancer activity against pancreatic cancer cells and pancreatic cancer stem cells was evaluated in vitro. The present study revealed that the novel agent decreases the proliferation of PDAC cells. These actions are mediated at the level of the mitochondrion by promoting ROS production and activating the ER stress pathway and autophagy. Taken together, TPP-PA has emerged as the prototype of a new class of anticancer agents that act via a novel chemical strategy to kill both “bulk” cancer cells and PCSCs. These findings suggest that TPP-PA has potential value in the treatment of PDAC.

## Figures and Tables

**Table 1:** Cardiolipin content in P and PCSCs after TPP-PA treatment (18 μM, 24 h).

	P		PCSCs	
	Control	TPP-PA	Control	TPP-PA
Cardiolipin (nmol/mg protein)	49 ± 8	42 ± 1	55 ± 6	62 ± 7

## Data Availability

All the research data are shared in this manuscript.

## Supplementary Materials

The following supporting information can be downloaded at https://www.mdpi.com/article/10.3390/ijms25126790/s1.
